# Neonatal Screening for Sickle Cell Disease in Congo

**DOI:** 10.1155/2022/9970315

**Published:** 2022-02-03

**Authors:** Alexis Elira Dokekias, Lethso Thibaut Ocko Gokaba, Josué Simo Louokdom, Lydie Ngolet Ocini, Firmine Olivia Galiba Atipo Tsiba, Coreillia Irène Ondzotto Ibatta, Quentin Ngoma Kouandzi, Serge Talomg Tamekue, Jayne Chelsea Bango, Jade Vanessa Nziengui Mboumba, Simon Charles Kobawila

**Affiliations:** ^1^Centre National de Référence de la Drépanocytose ‘' Antoinette SASSOU N'GUESSO, Brazzaville, Congo; ^2^Université Marien Ngouabi, Brazzaville, Congo

## Abstract

**Introduction:**

Sickle cell disease is an autosomal recessive inherited disorder due to the mutation of a gene coding for the globin beta chain. The aim of this study is to update the epidemiological data on hemoglobinoses, in particular sickle cell disease in newborns in Congo.

**Materials and Methods:**

This was a descriptive cross-sectional study, conducted from October 1, 2019, to March 31, 2020, throughout the Congolese national territory. It involved all full-term newborns, without distinction of nationality, aged 5 days or less, and whose parents consented to participate in the study. The blood samples, taken at the heel and collected on Whatman blotting paper, were analyzed using the HPLC Variant NBS machine.

**Results:**

In 2897 newborns (NN) screened, hemoglobin abnormalities were found in 603 NN (20.81%). The mean age of these newborns was 1 day (extremes 0 and 5 days). The male-to-female ratio was 1.03. Abnormal hemoglobins were mainly Hb S (*n* = 597 (97.71%)); Hb C (*n* = 5 (0.82%)); and variants (*n* = 7 (1.15%)). The national prevalence of major sickle cell (MSC) syndromes and sickle cell trait was 1.35% and 19.43%, respectively. The prevalence ranged from 1.77% to 2.56% for MSS in four departments and from 20.5% to 25.8% for the sickle cell trait in six other departments.

**Conclusion:**

Data on homozygous sickle cell disease remain consistent with previous studies. However, further studies should clarify the molecular anomalies of the variants observed in our samples.

## 1. Introduction

Sickle cell disease is an autosomal recessive inherited disorder linked to the mutation of a gene coding for the beta chain of globin [1–3].

It is the first genetic disease in the world which mainly comprises four (4) primitive foci: African focus, Asian Peninsula, South-East Asia, and an incidental focus in the Balkans.

A distinction is made between heterozygous forms (AS) and homozygous forms (SS) which are part of the major sickle cell syndromes (MSS). The major sickle cell syndromes include the following entities: homozygous abnormal hemoglobin S, hemoglobin S associated with other abnormalities (C, D, and O), and intermediate forms of thalassemia. Multiple studies have been carried out worldwide on the prevalence of this genetic disorder [4–9]. In Africa, studies report a prevalence of 0.8 to 3% of major forms and 7 to 24% of heterozygous forms [4, 5, 10–16]. Studies in Congo have involved local sampling and analysis, most often carried out outside Congo [4, 16]. These analyses placed the frequency of sickle cell trait in Congo between 19 and 24% and the homozygous form around 1% according to neonatal samples [4, 16].

This study, which focuses on neonatal sampling, was conducted entirely in Congo. The samples were taken from newborns in all departments of the Republic of Congo, and the analyses were carried out on site. In order to update the data on sickle cell disease in Congo, we conducted this study to determine the prevalence of sickle cell disease and to estimate the variants of hemoglobin (Hb) in newborns in each department of Congo.

## 2. Materials and Methods

**Figure 1:**
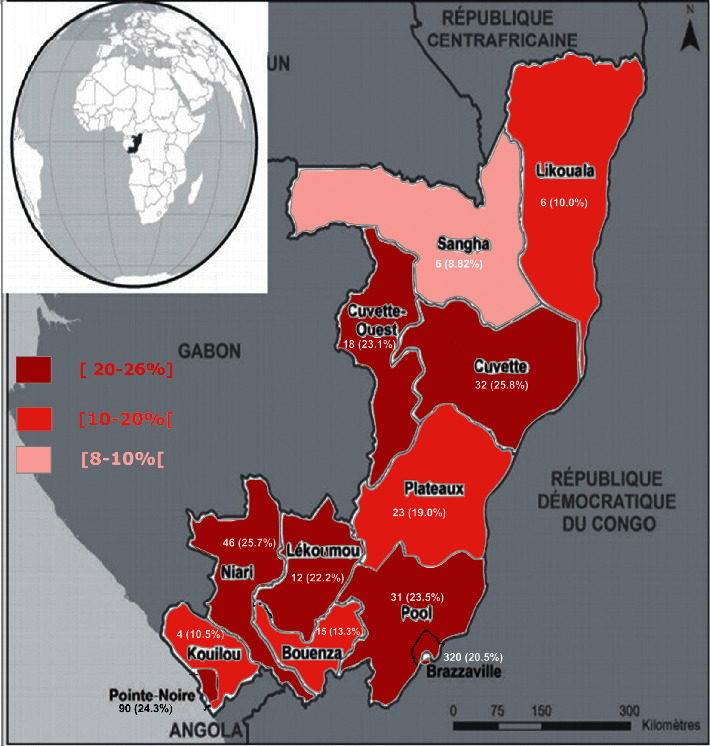
Prevalence of abnormal hemoglobin in newborns in the departments of Congo.

This was a cross-sectional descriptive study conducted from 1 October 2019 to 31 March 2020 over the entire national territory of Congo (Figure 1). The Republic of Congo is located in sub-Saharan Africa, in the sickler belt. It is made up of 12 departments (Figure 1) in which samples were collected and sent to the Centre National de Reference de la drepanocytose (CNRDr) for analysis. The CNRDr is a subregional facility, open to the public in 2016, specialised in the management of genetic diseases including sickle cell disease. It comprises care, imaging, and medical biology units.

The target population consists of full-term newborn babies (NBs) born in the Congolese territory. All NBs without distinction of nationality, aged 5 days or less, were included in the study. We did not include all NBs in the posttransfusion period and those in intensive care. Furthermore, only the NBs whose parents had given their informed consent were included. Only those whose parents gave consent were screened. Refusal to screen a newborn was a criterion of exclusion. The recruitment was carried out in a comprehensive way.

Considering the prevalence of the sickle cell trait of 21% [17] and the effect of the sampling design set at 1.5 and the imponderables estimated at 5%, the minimum sample size required for this study is 406. The number 406 corresponds to the minimum of patients needed to carry-on the study. It is not the sample of our study but the minimum required. The minimum sample size for each department (cluster) was calculated by weighting the national minimum baseline sample size (406 NBs) according to the size of the population in the department. By rounding up these numbers, the minimum sample size selected was 412, broken down by department (Table 1)

For each newborn baby, a drop of blood was collected at the heel on Whatman 903TM (903 protein saver card) blotting paper under aseptic conditions. The samples were stored for a maximum of 72 hours in pouches (Multi-Barrier Pouches) with desiccant from GE Healthcare company laboratories. They were analyzed using the cation exchange high-performance liquid chromatography (HPLC) procedure (BioRad Variant NBS). Patient profiles were obtained by ranking hemoglobins in descending order of magnitude (thus, an FSA profile assumes a hemoglobin F concentration greater than hemoglobin S concentration and the latter greater than hemoglobin A concentration). The chromatographic profile of MSS was established in the presence of Hb: FS, FSC, FSE, FSD, FSA, FSE, and FSa.

The data obtained were entered and processed using Microsoft Excel version 17.2 and R 3.6.3 software [18]. Qualitative variables were presented as numbers and percentages. Ethical clearance was obtained from the Comité Ethique de Recherche en Science de la Santé (CERSSA).

## 3. Results

At the end of this study, we obtained a total of 2,897 newborns, 603 of whom had abnormal hemoglobins, being 20.81%. The mean age of these newborns was one (01) day with extremes ranging from 0 to 5 days and a sex ratio (F/M) of 1.03. They were born at 38 (q1 36–q3 39) weeks of amenorrhea with an average weight of 3114 ± 567 g.

Figure 1 illustrates the prevalence of abnormal hemoglobin in newborns by department.

The abnormal hemoglobins found were Hb S (*n* = 597; 97.71%), Hb C (*n* = 5; 0.82%), Hb D (*n* = 1; 0.16%), Hb E (*n* = 1; 0.16%), Hb Bart (*n* = 1; 0.16%), and unidentified Hb (*n* = 7; 1.15%). The distribution of the different types of abnormal hemoglobin by department is described in Table 2.

Among the 2,897 newborns registered, major sickle cell syndrome (MSS) was found in 39 NBs and the sickle cell trait was found in 563, representing national prevalences of 1.35% and 19.43%, respectively (Table 3).

Heterozygous qualitative hemoglobin abnormalities were AS (*n* = 551 (97.87%)) and AC (*n* = 5 (0.89%)).

Table 4 reports the prevalence of the sickle cell trait and MSS phenotypes according to the departments of Congo.

## 4. Discussion

This study's objective was to update the epidemiological data on sickle cell disease in Congo. The systematic screening approach was favored over the approach targeting NBs from parents with a family history of sickle cell disease, firstly, because healthy AS carriers do not express the disease and are often unaware of their status, which makes it difficult to identify subjects at risk and, secondly, because of the high prevalence of the S gene in the Congo Basin. This screening was carried out using the HPLC variant NBS technique, which has the advantage of being fully automated but also has a high sensitivity to detect normal neonatal hemoglobins (Hb F and Hb A) and the main hemoglobin variants (Hb S, C, E, and D).

In our study, the sex ratio (1.03), in favour of men, was similar to that reported in the literature [5, 13, 14, 19–21]. The average weight of newborns with abnormal hemoglobins was comparable to observations made by Munyanganizi in Rwanda and Kafando in Burkina Faso [10, 19]. It was lower than the one reported by McGann in Angola, which found an average weight of 3.201 ± 526 g [15]. Dietary, environmental, and genetic factors could explain these differences.

The prevalence of abnormal hemoglobin was 20.8%, mainly represented by hemoglobin S (97.71%). The geographical location of the Republic of Congo in the sickler belt and the endemic nature of malaria in Congo could be one of the factors favoring this condition. Indeed, in order to better resist malaria, the populations of this region have undergone mutations that have led to the appearance of hemoglobin S. Subjects carrying this mutation in its heterozygous form present a certain resistance to *Plasmodium falciparum* 22. A selective pressure with the evolution of the disease would have contributed to the increase in its prevalence. Results found in the literature indicate abnormal hemoglobin frequencies ranging from 0.22% to 31% [4, 5, 9, 13, 20, 23–25]. Some of these observations are comparable to ours [4, 5]; on the other hand, others had different results from ours [9, 13, 20, 23–25]. Results comparable to ours can be justified by the fact that these studies were carried out in Central Africa in the Congo Basin, the epicenter of sickle cell disease as in our case [4, 5, 22]. The results differing from ours can be explained by the fact that these screenings were carried out in nonendemic or low-malaria-endemic areas where sickle cell disease cases are mainly imported cases or from families with relatives from highly endemic areas. This justifies the need for the establishment of a national program of systematic screening for sickle cell anemia in order to provide early management of newborns living with sickle cell anemia. Moreover, early management makes it possible not only to delay the onset of complications but also to undertake genetic counseling of the parents of newborn babies living with sickle cell anemia [25–27].

Sickle cell trait prevalences of around 25% were obtained in some departments (Cuvette Centrale, Niari, and Pointe Noire) and 10% in others (Sangha, Likouala, Kouilou, and Bouendza). The low prevalence in these regions can be justified not only by their relatively small numbers compared to those of other departments but also by cultural habits regarding marriage.

The main abnormal Hb encountered was Hb S (97.71%) and C (0.82%). Variant C was found only in NBs with Malian parentage. This observation is similar to that made by some authors [6, 13, 14, 20, 24, 28], but differs from others in West Africa where Hb C predominates in most series 10. Indeed, Hb C is the most common abnormal Hb in West Africa and more particularly in Burkina Faso where it originated [29].

The prevalence of MSS found in our study was higher than that of our Congolese predecessors (1.35% versus 1%). The increase in prevalence is explained by the fact that 2010 study [4] was conducted in only two cities, whereas this study covered the entire country. Furthermore, the MSS profiles observed in our study were FS, FSE, and FSA, whereas they reported only one hemoglobin profile (FS). The type of equipment used in 2010 and the criteria used to define MSS could justify these differences.

In addition, MSS prevalences ranging from 0.06% to 3% have been reported in Europe, South America, Central Africa, and other countries. These variations could be justified not only by the fact that MSS is endemic in some regions while in others, it is mainly imported cases but also by the lack of knowledge of sickle cell disease in regions of high prevalence and the existence of consanguineous marriages [5–7, 9, 11, 12, 14, 15, 21, 23, 24, 28].

Similarly, the prevalence of MSS is heterogeneous within the departments of Congo. It predominates in some departments where its prevalence is higher than that observed at the national level (Table 3). Cultural habits in terms of choice of spouse, the lack of systematization of premarital screening, the mere availability of the Emmel test, and the lack of awareness in some departments could explain this disparity and, hence, the interest in setting up a capacity-building system for awareness raising, screening of couples at risk, and even genetic counseling in order to help reduce the prevalence of sickle cell disease. In addition, it will make it possible to initiate prophylaxis with penicillin therapy, which substantially reduces the morbidity and mortality of NBs [27, 30].

With regard to the sickle cell trait, as in the study by Mpemba et al., its national prevalence is 19.43%. It varies from one department to another (Table 3). In addition, we observed that the department of Cuvette Centrale, although it has a high prevalence of sickle cell trait, has fewer cases of MSS (Table 3). These facts could be justified by the health education of the population in the department. Because Cuvette Centrale is a department with an area of 48,250 km^2^ and three general hospitals within a radius of 100 km, consanguineous marriage is prohibited. The prevalence of the sickle cell trait in our study is similar to that of some authors, who reported a prevalence of the sickle cell trait in a range of 16 to 21%. This could be justified by the fact that these countries belong to the Central African zone [4, 5, 14, 15]. In contrast, other authors in West and East Africa reported lower prevalences than ours (5 to 12%). This may be justified by the fact that these areas are far from the epicenter of sickle cell disease, which is in Central Africa [6, 10, 12, 13, 28]. Much lower prevalences (0.08 to 3.8%) are reported in European and American studies [9, 21, 23–25, 31].

In light of our observations, screening for sickle cell disease should also be systematic upstream during the premarital check-up in order to provide couples at risk with appropriate genetic counseling to guide their choice. However, it would be advisable to supplement this study with molecular biology techniques which would provide additional information on the possible presence of other haplotypes in Congo due to migration flows.

## 5. Conclusions

This work made it possible not only to update data on sickle cell disease in the neonatal period but also to provide epidemiological characteristics within each department of Congo. Prevalence remains stationary for the sickle cell trait, while it is increasing for major sickle cell syndromes. This resumes the frequency of homozygous and heterozygous forms of sickle cell disease in the Republic of Congo. The increase can be explained by the existence of consanguineous marriages and the absence of HbS screening tests in some regions, as well as the use of the Emmel test in other departments of Congo, although this test is less reliable The implementation of a national program leading to systematic neonatal screening will make it possible to identify and provide early care for newborns with homozygous forms of hemoglobin S, which is a guarantee of a better quality of life, by limiting infectious and vaso-occlusive complications. The study was conducted with the aim of making neonatal screening systematic. It is a project that needs funding to be sustainable.

## Figures and Tables

**Table 1:** Minimum sample size by department.

Department	Population census of 2007	Minimum newborn size to be tested	Number of newborns tested
Brazzaville	1733271	151	1560
Pointe Noire	902828	79	370
Bouenza	390032	34	113
Pool	298580	26	132
Niari	291865	26	179
Plateaux	220328	20	121
Cuvette	196930	18	124
Likouala	194507	17	60
Lekoumou	121639	11	54
Kouilou	116065	11	38
Sangha	108211	10	68
Cuvette-Ouest	92123	9	78
Total	4666379	412	2897

**Table 2:** Distribution of abnormal hemoglobins (Hb) in NBs by department between November 2019 and March 2020.

Department	HbS, *n* (%)	Autre Hb^*∗*^, *n* (%)	HbC, *n* (%)	HbE, *n* (%)	HbBart, *n* (%)	HbD, *n* (%)
Bouendza	15 (2.5)	2 (28.6)	—	—	—	1 (100)
Brazzaville	314 (52.6)	1 (14.3)	5 (100)	—	1 (100)	—
Cuvette Centrale	32 (5.4)	1 (14.3)	—	—	—	—
Cuvette-Ouest	18 (3.0)	—	—	—	—	—
Kouilou	4 (0.7)	—	—	—	—	—
Lekoumou	12 (2.0)	—	—	—	—	—
Likouala	6 (1.0)	—	—	—	—	—
Niari	46 (7.7)	2 (28.6)	—	—	—	—
Plateau	23 (3.9)	—	—	—	—	—
Pointe Noire	90 (15.1)	—	—	1 (100)	—	—
Pool	31 (5.2)	1 (14.3)	—	—	—	—
Sangha	6 (1.0)	—	—	—	—	—
Total	597 (100)	7(100)	5 (100)	1 (100)	1 (100)	1 (100)

*n* : number; ^*∗*^: unidentified Hb variant.

**Table 3:** Prevalence of sickle cell disease among newborns according to the departments of Congo between November 2019 and March 2020.

Department	Total, *n* (%)	Carrier Hb AA, *n* (%)	Carrier Hb AS/AC, *n* (%)	MSS^*∗*^, *n* (%)
Bouendza	113 (3.90)	98 (86.7)	13 (11.5)	2 (1.77)
Brazzaville	1560 (53.8)	1241 (79.6)	298 (19.1)	21 (1.35)
Cuvette Centrale	124 (4.28)	92 (74.2)	32 (25.8)	—
Cuvette-Ouest	78 (2.69)	60 (76.9)	16 (20.5)	2 (2.56)
Kouilou	38 (1.31)	34 (89.5)	4 (10.5)	—
Lekoumou	54 (1.86)	42 (77.8)	12 (22.2)	—
Likouala	60 (2.07)	54 (90.0)	6 (10.0)	—
Niari	179 (6.18)	133 (74.3)	44 (24.6)	2 (1.12)
Plateau	121 (4.18)	98 (81.0)	22 (18.2)	1 (0.83)
Pointe Noire	370 (12.8)	280 (75.7)	81 (21.9)	9 (2.43)
Pool	132 (4.56)	101 (76.5)	29 (22.0)	2 (1.52)
Sangha	68 (2.35)	62 (91.2)	6 (8.82)	—
Total	2897 (100)	2295 (79.21)	563 (19.43)	39 (1.35)

^*∗*^MSS: major sickle cell syndrome (FS, FSE, and FSA).

**Table 4:** Distribution of abnormal Hb chromatographic profiles in newborns according to departments between November 2019 and March 2020.

Department FAS^*∗*^	Heterozygous profile		MSS profile		
		FAC, *n* (%)	FS, *n* (%)	FSA^*∗∗*^, *n* (%)	FSE, *n* (%)
Bouendza	13 (100)	—	1 (50)	1 (50)	—
Brazzaville	293 (98.32)	5 (1.68)	8 (38.1)	13 (61.9)	—
Cuvette Centrale	32 (100)	—	—	—	—
Cuvette-Ouest	16 (100)	—	—	2 (100)	—
Kouilou	4 (100)	—	—	—	—
Lekoumou	12 (100)	—	—	—	—
Likouala	6 (100)	—	—	—	—
Niari	44 (100)	—	1 (50)	1 (50)	—
Plateau	22 (100)	—	—	1 (100)	—
Pointe Noire	81 (100)	—	4 (44.44)	4 (44.44)	1 (11.11)
Pool	29 (100)	—	—	2 (100)	—
Sangha	6 (100)	—	—	—	—
Total	558	5	14	24	1

^*∗*^6 cases of unidentified Hb variant and 1 Hb FASD; ^*∗∗*^FSA and FSa profile.

## Data Availability

Data supporting the results of this study can be found in the attached document.
